# Molecular Mechanisms of Cutaneous Inflammatory Disorder: Atopic Dermatitis

**DOI:** 10.3390/ijms17081234

**Published:** 2016-07-30

**Authors:** Jung Eun Kim, Jong Sic Kim, Dae Ho Cho, Hyun Jeong Park

**Affiliations:** 1 Department of Dermatology, St. Paul’s Hospital, College of Medicine, The Catholic University of Korea, Seoul 02559, Korea; mdkjeun@naver.com; 2 Department of Dermatology, Yeouido St. Mary’s Hospital, College of Medicine, The Catholic University of Korea, 62 Yeouido-dong, Yeongdeungpo-gu, Seoul 07345, Korea; rodmann@nate.com; 3 Department of Life Science, SookmyungWomen’s University, Seoul 140-742, Korea; cdhkor@sookmyung.ac.kr

**Keywords:** atopic dermatitis, genetics, epigenomics, barrier, immunologic abnormalities

## Abstract

Atopic dermatitis (AD) is a multifactorial inflammatory skin disease resulting from interactions between genetic susceptibility and environmental factors. The pathogenesis of AD is poorly understood, and the treatment of recalcitrant AD is still challenging. There is accumulating evidence for new gene polymorphisms related to the epidermal barrier function and innate and adaptive immunity in patients with AD. Newly-found T cells and dendritic cell subsets, cytokines, chemokines and signaling pathways have extended our understanding of the molecular pathomechanism underlying AD. Genetic changes caused by environmental factors have been shown to contribute to the pathogenesis of AD. We herein present a review of the genetics, epigenetics, barrier dysfunction and immunological abnormalities in AD with a focus on updated molecular biology.

## 1. Introduction

Atopic dermatitis (AD) is a common chronic inflammatory skin disease. The prevalence of AD in children is about 10%–20%, while the prevalence in adults is approximately 1%–3% worldwide, depending on the different ethnic populations [1]. It is known that most of AD-related genes do not follow Mendelian law, but are highly heritable. Thus, patients with familial history of AD have a higher risk of developing AD [2]. The prevalence of AD is higher in developed countries, such as those in Western Europe, and much lower in the countries specialized in agriculture, including China and Eastern Europe, rural Africa and Central Asia. This trend is consistent with the hygiene hypothesis [3]. Additionally, AD patients have various triggering factors and disease courses, which emphasize the influence of inter-individual differences. About 70% of patients with AD show elevated serum IgE levels with allergic sensitization and are categorized as having extrinsic AD, while other patients showing AD lesions with normal serum IgE levels are categorized as having intrinsic AD, although both subtypes share common clinical features [4]. Lesional distribution patterns vary depending on the patient’s age and disease activity. Whereas food allergens are the main triggering factors in pediatric AD, inhalant allergens are the main cause of AD exacerbation in adults [5]. The symptoms subside in many pediatric patients as they grow. However, some patients show persistent disease courses and tend to have concomitant allergic diseases, such as allergic rhinitis and asthma [6]. Generally diagnosis is made based on relevant clinical history and symptoms of the patients. The key clinical features of AD are pruritus and chronically-relapsing eczematous dermatitis that has a typical morphology and distribution distinct to individual age. These features can be used to distinguish AD from other clinical conditions, such as psoriasis and seborrheic dermatitis. Among various diagnostic criteria, Hanifin–Rajka’s criteria have been widely used [7]. The treatment strategy of AD mainly depends on the disease severity. At any stage, moisturizer should be properly used, and during flare-ups, topical and/or systemic immunomodulators can be used to control the conditions with different disease severity. The objective SCORing AD (SCORAD) index is widely used to assess AD severity [8]. Mild AD (SCORAD < 15) can be controlled by using moisturizer and topical anti-inflammatory agents, including topical steroids and calcineurin inhibitors [9]. The main treatment option of moderate (15 ≤ SCORAD < 40) to severe AD (SCORAD ≥ 40) includes systemic immunosuppressants, such as steroids, cyclosporine, azathioprine, methotrexate, interferon-gamma (INF-γ), intravenous immunoglobulin, allergen-specific immunotherapy and phototherapy. The known indication for the above therapies is the AD patients with sleep disorders, emotional stress or SCORAD index >40. Antihistamines and antimicrobial drugs can be used if needed. Although biologics have been tried to treat AD, the efficacy is limited when compared to that of psoriasis. Adjunctive treatment, such as evening primrose, probiotics and alternative medicines, can be tried in refractory AD patients [9]. However, the ultimate outcome of current treatment modalities is often not satisfactory in severe AD patients and has significant side effects. There is accumulating evidence that the heterogeneity of AD may result from the complex interactions between genetic susceptibility and environment, resulting in decreased skin barrier function, defects in innate immunity and aberrant immune responses to allergens and pathogens. This information offers the potential for individually-tailored therapeutic approaches. Genome-wide association studies (GWAS) and Immunochip analyses have identified several gene polymorphisms, susceptibility loci for AD and genetic changes caused by environmental factors that may be involved in the pathogenesis of AD. Newly-found T cells and dendritic cell (DC) subsets, cytokines, chemokines and signaling pathways have extended our understanding of the molecular pathomechanism of AD and modified the conventional concept of T helper type 1 (Th1)/T helper type 2 (Th2) imbalance paradigms [10,11,12,13]. Recent advances in the understanding of the pathomechanism of AD regarding the barrier dysfunction and immune dysregulation have led to the development of new therapeutic drugs of AD, and the efficacy and safety of these drugs are currently under the investigation. This review summarized the updated pathogenesis of AD with regard to genetics, epigenetics, epidermal barrier disruption and immunological dysregulation.

## 2. Genetics

Many barrier and immune molecules related to the pathogenesis of AD showed potential genetic polymorphisms that may serve as protective or risk factors for AD (Table 1). Genes associated with epidermal barrier constituents and enzymes that maintain homeostasis have been linked to AD. Filaggrin gene (*FLG*) mutations are found in 10%–50% of patients with AD, but also in 9% of the normal population [14,15,16]. Loss-of-function mutations in *FLG* are well-known predisposing factors for AD and account for 40% of the total mutations in these patients. Whereas the frequency of null mutations of *FLG* is reportedly about 50% in European patients with moderate to severe AD, this frequency is much lower (<27%) in Asian patients with AD [17,18,19]. Only about 15% of patients with mild to moderate AD show the null mutation of FLG, even in European populations [17]. Therefore, FLG deficiency alone seems insufficient to explain epidermal barrier dysfunction in these patients. In addition to various mutations, copy number variation within FLG also increases the risk of AD [20,21], and the levels of filaggrin degradation products in the stratum corneum are correlated with the FLG genotype or copy number and AD severity [21,22]. Hornerin and FLG2 are fused S-100 proteins that are functionally related to FLG. Among patients with FLG2 mutations, African-Americans tend to develop more persistent AD [23]. A single-nucleotide polymorphism (SNP) in epidermal differentiation complex (EDC) on chromosome 1 and variants of C11orf30 and LRRC32 on chromosome 11 were found to be associated with AD [24]. Small proline-rich protein 3 (SPRR3) is a cornified envelope (CE) precursor protein. Several mutations related to SPRR3 overexpression in patients with AD are associated with decreased production of lipid levels and a thinner CE [25,26]. Mattrin encoded by transmembrane protein 79 (TMEM79) regulates lamellar body secretion. The missense mutation rs6684514 of TMEM79 reportedly impairs lamellar body secretion in patients with AD [27,28,29]. Serine protease inhibitor Kazal-type 5 (SPINK5) encodes the protease inhibitor lymphoepithelial Kazal-type-related inhibitor (LEKTI), which counteracts the activity of epidermal proteases, such as kallikrein-5 in the epidermis. Loss-of-function mutations in SPINK5 are known to cause Netherton syndrome, which has AD manifestations. The rs2303070 T allele of SPINK5 is a risk factor for AD in Taiwanese populations [30]. The E420K LEKTI variant is associated with an increased risk of developing AD through increased TSLP expression and barrier permeability by enhancement of epidermal protease activity and profilaggrin proteolysis [31]. The claudins are key adhesion molecules comprising tight junctions. The claudin-1 (*CLDN1*) gene haplotype-tagging SNP reportedly has associations with AD in North American populations [32]. The rs9290929 polymorphism, located in *CLDN1*, reduced the expression of *CLDN1* and enhanced the production of IgE after mold exposure [33].

Several gene polymorphisms related to innate and adaptive immunity have been found in patients with AD. These include mutations in pathogen-associated molecular patterns, such as toll-like receptor (TLR) and nucleotide-binding oligomerization domain receptors (NOD) and antimicrobial peptides (AMPs), TSLP and the receptor for TSLP (TSLPR), IL-1 family cytokines and receptor genes, vitamin D pathway genes, the nerve growth factor pathway, Th2 and other cytokine genes and the genes encoding the high affinity IgE receptor (FcεRI, FCER1A) [24,34,35,36,37,38,39]. About 12% of patients with AD have the (TLR)2 R753Q mutation, and this mutation is associated with the severe AD phenotype and concomitant atopic diseases in certain populations [40,41,42,43]. TLR2 A-16934T, TLR4 D299G and A-896G mutations are also associated with severe AD [43,44]. The TLR9 promoter polymorphism, C-1237T, has been reported in patients with intrinsic AD [45]. SNPs in NOD-like receptor 1 genes related to caspase recruitment domain (CARD)4, CARD12, CARD15, NACHT, LRR and PYD domain-containing protein (NALP)1, NALP12 and NOD1 have been associated with AD [7,46]. Several SNPs of the human β-defensin (*hBD*) 1 gene were found to be linked to severe AD with allergic sensitization [47]. TSLP plays a crucial role in DC-driven Th2 responses. A recent study showed that patients with AD with a certain TSLP polymorphism showed eczema herpeticum [48,49]. IL-1 family cytokines play important roles in innate immune responses in patients with AD. While some variants of the *IL-18* gene and the receptors (IL18RA) are associated with AD [35,50], rs1946518 and rs187238 polymorphisms in the *IL-18* gene were shown to be the protective factors against the development of AD [51]. Genetic variants in *IL-12* and *IL-12RB*, *IFN-γ* genes (IFNG) and IFNGR1 leading to partial IFNGR1 deficiency are related to AD in patients susceptible to eczema herpeticum [52,53]. Whereas CYP27A1 variants in the vitamin D pathway genes showed a protective effect, other variants are associated with severe AD with eosinophilia and high IgE levels [35,54,55,56].

Several distinct polymorphisms of IL-4, IL-13 and IL-31 and their receptors were found to influence AD predisposition [34]. Genetic differences in the genes encoding IL-4 and IL-13 were suggested to alter transcriptional activity. The signal transducer and activator of transcription 6 (STAT6) is a key transcription factor in responses mediated by IL-4 and IL-13. STAT6 variants were found to be associated with AD [57,58]. Neonates with the rs324011 polymorphism in the STAT6 had a lower risk of AD as they showed a reduced number of regulatory T cells (Tregs) and an increased Th1 response at birth [59]. A common haplotype encoding IL-31 was shown to be a risk factor for intrinsic AD. The rs7977932 G allele of IL31 variants was shown to be a risk factor for AD in Taiwanese populations [30]. The haplotype AAA or GAA of IL-31 was correlated with increased serum levels of IL-31 and severe pruritus in certain populations [60]. The AA genotype of IL-17A was found to be a predisposing factor of severe AD with concomitant asthma [61]. Other cytokine variants were also identified in patients with AD, including IL-2, IL-5, IL-6, IL-7, IL-9 and IL-10 [50,53,62,63,64,65,66,67,68]. Polymorphisms of regulated on activation, normal T cell expressed and secreted (RANTES) and eotaxin were associated with allergen sensitization [69,70,71]. A haplotype variant of the histamine 4 receptor (H4R) and a copy number variation were found to be associated with AD [72,73]. An SNP of FCER1A was reported to be associated with AD in Asians with an elevated serum IgE level [74,75,76]. The T allele of brain-derived neurotrophic factor gene polymorphism in C270T is associated with intrinsic AD and male sex. Serum brain-derived neurotrophic factor levels were reported to be correlated with the severity of intrinsic AD [77].

Barrier strengthening therapy to improve the barrier defect can be achieved by the proper use of moisturizer. Biologics treatment targeting Th2 immunity could be the best therapeutic option in the future. Duplimumab, anti-IL-4Ra monoclonal antibodies (mAb), has shown promising therapeutic responses in phase III clinical trials [78]. Anti-IL-13 mAb (lebrikizumab and tralokinumab) is currently in phase II clinical trials for AD [79]. Anti-IL-22 mAb (ILV-094), anti-IL-31 (BMS-981164) and anti-IL-31R (CIM331) mAb, anti-TSLP (AMG 157) and anti-TSLPR (MK-8226) mAb have been developed and are currently in phase II, I, II, I and I clinical trials, respectively [80]. OC000459 and other several small molecules that antagonize the chemoattractant receptor-homologous molecule expressed on Th2 cells have been in a phase II clinical trial of AD patients [80].

## 3. Epigenetics

The modern lifestyle and other environmental factors, such as air pollutants and tobacco smoke, have been suggested to be responsible for the high prevalence of AD since the advent of industrialization. There is accumulating evidence that epigenetic changes in response to these environmental factors contribute to the pathogenesis of AD. These epigenetic mechanisms include DNA methylation, histone modification and microRNA (miR) responsible for barrier function and immunological regulation [81].

Prenatal tobacco smoke exposure is correlated with high-level miR-223 expression and DNA methylation of the FOXP3 locus in cord blood, which are associated with lower Treg numbers. Infants with lower Treg numbers in cord blood at birth had a higher risk of AD during the first three years of life [82,83]. The potential risks of AD are determined by both the amount of exposure to or composition of the pollutants and the genetic susceptibility of the host [84]. A Taiwanese birth cohort revealed that a gene polymorphism related to a deficiency of the antioxidant enzyme glutathione-*S*-transferase may explain the individual differences in susceptibility to AD after prenatal tobacco smoke exposure [85].

DNA demethylation of a specific regulatory region of the *TSLP* gene was significantly associated with TSLP overexpression in lesional skin of patients with AD [81]. Methylation of the TSLP 5′-CpG island was significantly linked with prenatal smoke exposure. The lower degree of such methylation in cord blood leads to *TSLP* overexpression and subsequent development of AD [86]. DNA methylation of genes related to FcεRI and IgE production may modify allergic sensitization in certain patients with AD. In AD-affected patients with high IgE levels, the levels of DNA cytosine methyltransferase 1 transcripts were significantly decreased in peripheral blood mononuclear cells [87]. Overexpression of FcεRI on monocytes and DCs in patients with AD was shown to be due to the demethylation of specific regions within the FCER1G locus [88].

Some miRs that are upregulated or downregulated in AD-induced lesions have been identified and involved in the pathogenesis of AD. Exposure to relevant allergens could induce miR-155 expression in AD lesions. Increased miR-155 downregulates cytotoxic T lymphocyte-associated antigen, a negative regulator of T cell function, which in turn stimulates T cell proliferation and leads to a sustained inflammatory state [89]. miR-155 also positively modulates the differentiation and function of T helper type 17 (Th17) cells and is correlated with AD severity [90]. Increased miR-146a expression has been reported in the lesional skin of patients with AD. miR-146a can alleviate AD inflammation by inhibiting nuclear factor κ B-mediated proinflammatory cytokines and chemokines [91]. The forced expression of miR-143 reversed IL-13-induced inhibition of epidermal differentiation by blocking IL-13Rα1. Thus, miR-143 may be a potential therapeutic target in AD [92]. However, evidence regarding epigenetics responsible for AD is still currently limited, and further studies are needed to clarify the gene–environmental interactions and potential therapeutic targets.

## 4. Barrier Dysfunction

Impaired epidermal barrier function in AD is characterized by abnormalities in skin microenvironment, gene functioning epidermal structural proteins, such as filaggrin and claudin, and lipid synthesis. The disrupted barrier causes an increased trans-epidermal water loss and enables the capture of more allergens, thus promoting allergic sensitization and initiation or exacerbation of AD inflammation. Impaired barrier function causes increased IL-1 release from keratinocytes, which activates the vascular endothelium to induce adhesion molecule expression and promotes cutaneous inflammation [12,93]. Epicutaneous sensitization to allergens causes heightened allergic immune responses and serves as a predisposing factor for a more severe allergic march.

The use of soap and detergent raises skin pH in AD patients, which induces the imbalance between serine proteases and protease inhibitors. The activities of endogenous and exogenous proteases from house dust mites or *S. aureus* are increased in AD lesions. Lack of endogenous protease inhibitor activity accelerates barrier permeability and inflammation. Increases in pH and in serine protease activity result in increased microbial colonization and accelerated degradation of the ceramide synthesis enzymes [94]. In a LEKTI-knockout mouse model, increased kallikrein-5 stimulated proteinase-associated receptor-2 (PAR2), which activates nuclear factor κ B-induced overexpression of thymic stromal lymphopoietin (TSLP) and induces pruritus [95]. TSLP also suppresses the expression of EDC proteins, such as filaggrin, by activating STAT3 and extracellular signal-regulated kinase (ERK) signaling in keratinocytes [96]. Phosphodiesterase 4 (PDE4), which catalyzes the conversion of cyclic adenosine 3′,5′-monophosphate (cAMP) to 5′-AMP, contributes to the pathogenesis of AD via PAR2. Anti-PDE4 agents could theoretically inhibit PAR2 and leukotriene B4 production mediated by increased cAMP levels, thus relieving pruritus in AD [97]. However, the oral anti-PDE4 apremilast and topical anti-PDE4 crisaborole showed limited efficacy in treating AD [98,99].

Filaggrin, a key protein in the skin barrier, is involved in cornification and hydration. FLG deficiency is known to increase [13] and impair skin integrity, hydration, protease activity and AMP function [12]. Reduced levels of hornerin and filaggrin-2 expression were suggested to be related to abnormal cornified envelope (CE) formation in AD skin [100]. In addition to filaggrin, loricrin is a component of CE, and decreased loricrin levels are observed in patients with AD. Filaggrin expression is restored after topical anti-inflammatory treatment with calcineurin inhibitors or corticosteroids [12,101]. A recent study demonstrated that JTC801, a new synthetic compound, increased filaggrin expression in human keratinocytes in vitro and decreased the development of AD-like lesions in mice in vivo [102]. The importance of barrier strengthening therapy is supported by the outcome that a topical recombinant filaggrin delivery through cell penetrating peptide could restore the AD-like inflammation in filaggrin knockout mice [103]. Defects of tight junction proteins, such as claudin, could have a permissive effect on the entry of irritants, allergens or pathogens into the epidermis [33,104]. The claudin-1 level showed an inverse correlation with a high serum IgE level and eosinophilia. Claudin-1 expression was significantly suppressed by IL-4, IL-13 and IL-31 in a human skin equivalent [105]. While claudin-1 knockout is lethal, low claudin-1-expressing conditioned mice exhibited AD-like dermatitis and an increased recruitment of neutrophil and macrophage in the skin. TLR2 activation increased the expression of tight junction proteins, including claudin-1, in human keratinocytes. TLR2-deficient mice showed slow and incomplete barrier recovery by suppressing the tight junction proteins [106]. Patients with AD show impaired skin integrity and subclinical inflammation even in uninvolved skin. Consistent with these features, markedly reduced levels of filaggrin, filaggrin-2 and claudin-1 expression were observed not only in lesional AD skin, but also in non-lesional skin in patients with AD [107].

Lipids in the stratum corneum comprise ceramides, free fatty acids (FFAs) and cholesterol. An overall reduction in lipid levels, especially in the ceramide content, and a reduced ceramide chain length are observed in patients with AD and are associated with the severity of AD [108]. A decrease in the FFA chain length and an increase in the proportion of unsaturated FFAs have been found in patients with AD [109]. A decreased lipid content leads to a less compact lipid organization and defective skin barrier function [109]. Th2-dominant cytokine profiles in AD further contribute to the decrease in ceramide and long-chain FFA levels [28,110].

## 5. Immunological Abnormalities

### 5.1. Innate Immunity

Patients with AD develop recurrent skin infections. Early studies suggested that suppressed levels of AMPs, such as hBD-2, hBD-3 and cathelicidin, in AD-affected skin compared to the skin of patients with psoriasis or healthy subjects, are responsible for this susceptibility to infection [111]. IL-4, IL-13, IL-10 and IL-33 could suppress the expression of hBD-2 and hBD-3 [112], contributing to superinfection [110]. In contrast, recent data showed that the levels of AMPs in AD lesions are increased as much as those in healthy subjects, but are still insufficient to defend against *S. aureus* infection, possibly because of the huge amount of *S. aureus* colonization or functional defects in the AMPs [113]. Members of the S100 protein family (S100A7, S100A8 and S100A9) function as AMPs, as well as damage-associated molecular pattern molecules, which have proinflammatory activities. The levels of S100 proteins are increased in patients with acute and chronic AD, and their proinflammatory properties may result in defects of epidermal differentiation and cutaneous inflammation [113,114].

TLRs play important roles in innate immunity by recognizing PAMP and antimicrobial defenses. The activation of TLRs induces the expression of antimicrobial effector molecules and the release of various proinflammatory and immunomodulatory cytokines, which lead to the activation of adaptive immune responses. In AD, enhanced Th2 cytokines were found to downregulate the expression of TLRs, which renders AD skin more susceptible to skin infections. Several TLR polymorphism have been reported to be associated with AD. Monocytes from patients with AD with the TLR2 R753Q mutation show enhanced IL-6 and IL-12 production and downregulated CD36 expression. These abnormalities cause impaired TLR2/TLR6 heterodimer-CD36 complex internalization, leading to increased susceptibility to *Staphylococcus aureus* (*S. aureus*) infection [115]. Vitamin D contributes to cathelicidin and lipid synthesis, and the efficacy of vitamin D supplementation has been suggested in small studies [116,117]. Several SNP polymorphism in vitamin D could affect TLR activity by inhibiting the cathelicidin expression [55,56].

The main sources of TSLP are epidermal keratinocytes, and the TSLP level is increased in the epidermis of AD lesions. TSLP is involved in the activation of Langerhans cells (LCs) and DCs to induce Th2 immune responses. TSLP showed a positive correlation with IL-31 and IL-33 and has been suggested as new biomarker in AD [118].

### 5.2. Adaptive Immunity

#### 5.2.1. Th1/Th2 Imbalance

Skin-homing memory T cells are key players of immune dysregulation in the pathogenesis of AD. Traditionally, the main pathogenesis of AD has been interpreted as immune dysregulation with predominant Th2 cytokines, such as IL-4, IL-5 and IL-13. As new T cell subsets were identified, the view of AD was modified as a Th2/Th22 polarized environment. Acute lesions of AD are driven by Th2 and Th22 responses, while chronic lesions are driven by a Th1 response. A recent study confirmed AD to be a result of Th2-skewed immune response, specifically the ratio of Th1:Th2 of chronic AD was 0.09. Consistently, most of the CD3^+^ T cells in biopsy specimens from chronic AD lesions were comprised of Th2 (64.6%), followed by Th17 (30.4%), Th22 (3.3%) and Th1 cells (4.8%) [119].

The Th2 cytokines IL-4 and IL-13 have a permissive effect on microbial invasion and epidermal barrier disruption by inhibiting AMP production [120], reducing lipid production in the stratum corneum and inducing spongiosis [80]. Th2 cytokines downregulate the expression of the major EDC genes, including FLG, LOR and involucrin [121,122,123,124], independent of the FLG genotype [125], and suppress keratinocyte differentiation via STAT3. Topical treatment with the Janus kinase (JAK) inhibitor downregulated STAT3 activation and restored skin barrier function by inducing terminal differentiation in AD animal models [126]. Topical and oral JAK inhibitors, such as tofacitinib, baricitinib and PF-04965842, are in phase II trials for AD [79]. IL-4 and IL-13 stimulate keratinocytes to express TSLP, which serves as a link between barrier defects and Th2 polarization. IL-4-overexpressing transgenic mice develop AD-like lesions. IL-5 attracts eosinophils into chronic AD lesions.

Th2 cytokines IL-4 promote immunoglobulin switching in B cells, resulting in IgE synthesis; induce the expression of adhesion molecules; and recruit various immune cells into skin. With regard to biological agents targeting the Th2 response, several clinical trials have confirmed the therapeutic efficacy and safety of the anti-IL-4 receptor antibody duplimumab [127].

Levels of the Th1 chemokine CCL20 are increased in patients with chronic AD [71]. Among the Th1 cytokines, IL-1α, IL-2 and TGF-β were found to be decreased in patients with AD [71], whereas IFN-γ, IL-12 and granulocyte monocyte-colony stimulating factor were elevated in patients with chronic AD. IFN-γ is known to activate keratinocytes and induce their apoptosis. Granulocyte monocyte-colony-stimulating factor is known to prolong the survival of monocytes and induce persistent inflammation. IL-11 and TGF-β1 are associated with tissue remodeling in chronic AD [128].

Treg cells are known to suppress both Th1 and Th2 immune responses and to be deficient in AD lesions.

#### 5.2.2. Th17 Cells

Th17 cells contribute to the onset of acute AD, although their role in AD is relatively small in psoriasis [12]. Th17 cells produce IL-17 and IL-22, which induce the production of S100 proteins, the AMPs in keratinocytes and various proinflammatory cytokines. IL-17 may have a role in the differentiation of Th2 cells [80]. IL-17 level decreases gradually in chronic AD lesions as Th2 cytokines inhibit IL-17 production. The relative absence of IL-17 in AD lesions may be related to reduced AMP levels and may explain the increased susceptibility to skin infection in patients with AD [129]. IL-17 is responsible for eosinophil and neutrophil-mediated inflammation [130].

The number of Th17 cells and IL-17 expressed in AD lesions and serum is correlated with disease severity [12,131], and this correlation is more prominent in intrinsic AD. A recent study comparing the transcriptome between intrinsic and extrinsic AD indicated that patients with intrinsic AD showed greater Th17 and Th22 immune activation than did those with extrinsic AD [132]. Compared to European and American AD patients, Asian patients presented more epidermal hyperplasia, parakeratosis and stronger Th17 and Th2 activation resembling psoriasis, even in patients with extrinsic AD [133]. However, another study in the European American population found that a number of Th17 cells and IL-17 expression level were reduced in the AD patients with severe symptoms, whereas those of Th2 and Th22 cell subsets were correlated with disease severity [134]. This discrepancy may result from the heterogeneous characteristics of the patients, such as phenotype, disease duration and ethnic differences.

#### 5.2.3. Th22 T Cells

The original concept of AD cannot account for the epidermal hyperplasia in patients with chronic AD and has some limitations. Th22 cells produce IL-22, which is responsible for skin barrier impairment and epidermal hyperplasia. Filaggrin expression can be modulated in AD by both the Th2 and Th22 cytokine milieu. Similar to Th2 cytokines, IL-22 compromises the epidermal barrier by suppressing major terminal differentiation proteins [135]. IL-22 increases S100A7, S100A8 and S100A9 gene expression, thus inhibiting epidermal differentiation by enhancing IL-6 secretion, and exerts a proinflammatory effect in AD lesions [114,121]. IL-22 and IL-17 synergistically increase the levels of S100 proteins and AMPs in the epidermis [12,136]. IL-22 secretion can be induced immediately in response to staphylococcal exotoxins and house dust mites and can potentially amplify chronic skin inflammation in patients with AD [137,138]. IL-22 can also stimulate CCL17 production from human keratinocytes and promote the migration of T cells into the skin [138]. Upregulated IL-22 in chronic AD induces matrix metalloproteinase-3, a marker of remodeling; stromelysin-1; platelet-derived growth factor A; and CXCL5 (chemokine, CXC motif, ligand 5) [139]. IL-22 promotes the migration of keratinocytes through matrix metalloproteinases-1 and -3 and is involved in epidermal hyperplasia [136].

IL-22 binds to its receptors in the form of an IL-22R1/IL-10R2 complex and activates the JAK-STAT signaling pathway via STAT3 activation [140,141]. IL-22 expression can be enhanced by the activated Notch signaling pathway without activation of STAT3 [142]. The role of Th22 cells varies depending on the patient’s age and disease severity. Infants with AD show only a Th2/Th1 cell imbalance, whereas adults with AD exhibit Th22/Tc22 cell subsets [143]. The numbers of Th22 cells are correlated with disease activity. Moreover, the skin-homing T cell population of patients with severe AD mostly comprises circulating Th2 and Th22 cells, rather than Th17 cells in European Americans [134]. The role of anti-IL-22 monoclonal antibody is currently being investigated in a phase II trial [80].

#### 5.2.4. IL-18

IL-18 is a Th1-like cytokine, which is produced by various cells. Keratinocytes and mast cells produce IL-18 in response to exposure to allergens or pathogens, such as house dust mites and *S. aureus* [144,145]. Cytotoxic T cells release perforin and granzyme B after the recognition of viral infection in keratinocytes, then subsequently activate pro-IL-18 [146]. Inflammatory dendritic epidermal cells (IDECs) and monocyte-derived DCs also release IL-18 [147]. IL-18 stimulates mast cells to release chymase, which in turn cleaves and activates pro-IL-18. IL-18 stimulates either Th2 or Th1 cytokines based on IL-12. In acute AD lesions, IL-18 stimulates basophils, mast cells and CD4^+^ T cells to produce Th2 cytokines without IL-12. In chronic AD lesions, IL-18 stimulates Th1 cells to produce IFN-γ with IL-12 [148]. IL-18 contributes to CD4^+^ T cell- and natural killer T cell-dependent IgE production [146,149]. Corticotrophin-releasing hormone, a skin hypothalamic-pituitary axis molecule, decreases IL-18 expression in monocyte-derived DCs independent of corticotropin-releasing hormone receptors in patients with AD [150]. This suggests that stress may aggravate AD symptoms by modulating the expression of IL-18.

IL-18 was shown to be a key player in the development of spontaneous AD-like lesions in a mouse model under pathogen-free conditions [151]. Recently, IL-18 levels in the maternal serum and cord blood were found to be a predisposing factor of childhood AD [152]. Elevated levels of serum IL-18 and IL-18 receptors have been suggested as biomarkers of AD severity [146,153], but additional research is required to confirm this suggestion [154,155].

#### 5.2.5. IL-31

IL-31, a new Th2 cytokine, is a major pruritogenic inflammatory substance in AD [156]. IL-31 protein and mRNA levels are elevated in AD lesions. Serum levels of IL-31 are proportional to disease severity in patients with AD [157]. IL-31 was found to inhibit epidermal terminal differentiation and enhance proinflammatory cytokine secretion [158,159]. IL-31 can also compromise epidermal barrier function by affecting epidermal terminal differentiation and lipid constituents, and it may inhibit terminal differentiation by downregulating filaggrin and loricrin expression [80,159] IL-31 also decreases ceramide production, increases long-chain FFAs, but decreases ester-linked ω-hydroxy ceramides in stratum corneum lipids [159].

IL-31 functions after binding to a heterodimeric receptor consisting of receptors for IL-31 (IL-31RA) and the oncostatin M receptor β complex. IL-31RA is found on keratinocytes, macrophages, eosinophils and nerve fibers in AD and in the neurons of dorsal root ganglia in healthy subjects [160,161]. IL-31/IL-31RA complexes activate signal transduction cascades, such as the Janus kinase-STAT (JAK-STAT), mitogen-activated protein kinase and phosphatidyl-inositol 3-kinase pathways [160]. The IL-31/IL-31RA complex stimulates the growth and branching of the nerve in sensory neurons via the STAT3 pathway and not via transient receptor potential cation channel vanilloid subtype 1 channels (TRPV1) [162].

A recent study demonstrated the safety and efficacy of a humanized anti-human IL-31RA monoclonal antibody in relieving pruritus in AD [163]. This suggests that the pruritogenic effect of IL-31 is mediated by IL-31RA. Activation of IL-31RA in keratinocytes induces calcium influx and produces more β-endorphin in STAT3-dependent pathways [164]. Meanwhile, a recent study suggested that the pruritogenic effect of IL-31 was mediated indirectly via keratinocytes and secondary pruritogenic substances, rather than through its receptors on cutaneous nerves [165]. Low doses of IL-31 promote the antimicrobial barrier, and thus, complete inhibition of IL-31 signaling may be undesirable [166].

#### 5.2.6. B cells

Although T cells are key players in the pathogenesis of AD, B cells are also found in the dermis of AD lesions and play significant roles. B cells can present antigens to CD4^+^ T cells and activate T cells. B cells interact with T cells via MHC class II molecule/T cell receptor and co-stimulatory molecules, as well as their ligands. Co-stimulatory molecules include CD40 and CD80/CD86 on B cells and their ligands, CD40L and CD28 on T cells. The expression of costimulatory molecule CD86 was reported to be increased on B cells in AD [167]. Th2 cytokine IL-4 promotes immunoglobulin switching in B cells, resulting in IgE synthesis, which then induces the expression of adhesion molecules and recruits various immune cells into skin. B cells also produce chemokines CCL17, CCL22 and IL-16, attracting T cells to AD lesions. The levels of IgE are significantly increased in AD patients along with allergic sensitization. IgE contributes to IgE-mediated inflammation by stimulating FcεRI-expressing cells, such as mast cells and basophils. Additionally, IgE also contributes to autoallergic inflammation in a certain subset of AD patients. Serum IgE autoantibodies to epidermal self-antigens, such as Homo sapiens antigens 1, had a 38.2% prevalence in AD patients of autoreactivity, while patients in the control group showed a 6.4% prevalence [168]. However, the role of IgE in pathogenesis of AD is not major, and previous attempts to inhibit IgE with omalizumab, respectively, showed heterogeneously different therapeutic efficacy in AD patients [169]. Although B cell-targeting therapy with rituximab (anti-CD20 antibody) showed some improvement in AD patients, it was conducted only in a case series [170].

#### 5.2.7. Dendritic Cells (DCs)

DCs play a key role in antigen uptake and presentation and induce a Th2-predominant response. Several types of DCs have been shown to express FcεRI in patients with AD [13]. These DCs are easily stimulated and activated by allergens, pathogens, irritants and scratching. Whereas LCs are the predominant DC type in non-lesional AD skin, inflammatory subtype DCs are increased in AD lesions [171]. IDECs have an increased capacity for antigen presentation and T cell activation. Topical anti-inflammatory treatment showed a reduced number of inflammatory DC subtypes in AD lesions [172]. On the other hand, the low number of plasmacytoid DCs and reduced amount of type 1 IFN in the lesional epidermis of patients with AD have been suggested to be responsible for the high susceptibility to viral skin infections in these patients [173].

TSLP is abundantly expressed in the epidermis of AD lesions. TSLP induces DCs to express OX40 ligand, which is essential for the differentiation of Th2 cells in the lymph nodes [174]. Activated DCs migrate to the lymph nodes and prime naive T cell differentiation into Th2 cells in the presence of IL-4 [174]. Expression of the IFN-γ receptor on epidermal DCs and their response to IFN-γ are attenuated in patients with AD [175]. DCs produce IL-25, which induces Th2 cell differentiation and suppresses filaggrin expression in vitro [12].

Activated epidermal LC and dermal DCs release CCL17 and CCL22, respectively, which in turn recruit and expand Th2 cells [176]. Chemokines (i.e., CCL17, CCL18, CCL19, CXCL9, CXCL10 and CXCL11) expressed by DCs induce further attraction and activation of various inflammatory cells [121]. Epidermal LCs and dermal DCs also induce the differentiation of Th22 cells, and IL-6 and TNF-α released from DCs have been suggested as contributing factors [177].

Plasmacytoid DCs also stimulate naive T cells to differentiate into Th22 cells in chronic AD lesions. IDECs release the proinflammatory cytokines IL-12 and IL-18, which promote Th1 responses in chronic AD. H4R is expressed by LCs, IDECs, plasmacytoid DCs and 6-sulfoLacnac (slan)-expressing DCs in patients with AD [178,179]. Histamine released from mast cells is relatively rich in AD lesions, subsequently leading to the activation of H4R-expressing DCs, and contributes to inflammatory processes.

#### 5.2.8. Chemokines

Thymus- and activation-regulated chemokine, also known as CCL17, is a key chemokine expressed on vascular endothelium and is involved in the homing of chemokine C-C motif receptor 4 (CCR4)-expressing T cells to the skin. CCL17 promotes the Th2 response via CCR4. The number of CCR4-expressing lymphocytes in serum is correlated with AD severity, the serum IgE level and the blood eosinophil count [180,181]. Furthermore, increased CCL17 levels in cord blood are associated with the development of infantile AD [182]. A recent meta-analysis indicated that serum CCL17 is the most reliable biomarker identified to date [154]. Similar to CCL17, macrophage-derived chemokine, also known as CCL22, is a chemoattractant for CCR4-expressing T cells. The Th2 chemokines CCL17 and CCL22 are mainly produced by LCs [183]. The levels of CCL17 and CCL22 may reflect the degree of skin barrier impairment. CCL17 and CCL22 induce the skin homing of T cells into AD lesions, and H4R antagonist inhibits CCL17 and CCL22 chemokine production by LCs in patients with AD [184]. Cutaneous T cell-attracting chemokine, also known as CCL27, is produced by keratinocytes and attracts CCR10-expressing Th22 cells into the skin. CCR6 is expressed in Th17 cells and facilitates their migration to the skin depending on the ligand CCL20 [185].

IFN-γ, monocyte chemotactic protein-4, eotaxin and RANTES secreted from keratinocytes after stimulation with Th1 cytokines facilitate the migration of macrophages, eosinophils and Th1 cells into chronic AD lesions [10]. CCL26 (eotaxin-3), which is essential to eosinophil recruitment into lesional epidermis, is enhanced by IL-4 and IL-13 [186]. IL-4 also stimulates dermal fibroblasts to express CCL11 (eotaxin-1) in AD lesions [187]. CCL11 was also detected on the lymphocytes, macrophages and eosinophils in AD lesions [188]. RANTES/CCL5 is a potent chemoattractant for eosinophils. CCL11, CCL5 and CCL26 bind to CCR3 expressed on eosinophils and activate these cells [189]. Fractalkine (CX3CL1) is expressed on the vascular endothelium and traffics leukocytes into AD lesions. Serum CX3CL1 levels were correlated with disease severity in pediatric AD patients [190,191].

#### 5.2.9. Mast Cells

The role of mast cells in the pathogenesis of AD is not completely understood. Increased numbers of mast cells are seen in AD lesions, especially in the chronic state. After exposure to allergens, FcεRI and IgE binding induce mast cell degranulation and cause acute symptoms by releasing preformed mediators, such as histamine, heparin, serotonin, prostaglandins, leukotrienes, major basic protein and platelet-activating factor. Patients with distinct STAT3 mutations present enhanced STAT3 signaling in mast cells and accelerated degranulation [192]. Histamine and tryptase released by mast cells evoke scratching behavior and secondary barrier disruption. Mast cells are involved in the pathogenesis of AD not only by releasing inflammatory mediators, but also by directly regulating the recruitment and action of various inflammatory cells. Mast cells regulate the differentiation, activation and migration of T cells by inducing the expression of chemokines and adhesion molecules on endothelial cells [193]. Prostaglandin D2, a mast cell mediator, downregulates IL-12 production by DCs, which leads to a Th2-polarized immune response [194]. Mast cells are among the sources of IL-4 and IL-13 production [195]. Overexpression of TSLP in AD lesions can further activate mast cells to generate more Th2 cytokines [196]. Ligands for CD40 on mast cells interact with CD40 on the surface of B cells and stimulate B cell development and IgE synthesis in the presence of IL-4 [197]. Histamine and TNF are known to facilitate the migration of DCs into lymph nodes [198,199]. Chemokines derived from mast cells attract eosinophils and type 2 innate lymphoid cells [10,200]. IL-33 can induce mast cells to produce proinflammatory cytokines and chemokines [201].

Mast cells not only serve as key inflammatory mediators, but also play a protective role in the development of AD. A recent study indicated that mast cell-knockout mice showed incomplete epidermal differentiation with decreased EDC gene expression and easily developed severe AD-like skin inflammation [202].

#### 5.2.10. Eosinophils and Basophils

Eosinophils are increased in number in both the serum and lesions of patients with AD, and they contribute to the pathogenesis of AD. Tissue eosinophilia is reportedly correlated with the severity of AD [203]. The Th2 cytokines IL-4, IL-5 and IL-13 play important roles in the development, survival, recruitment and function of eosinophils. Various inflammatory mediators are released during degranulation of eosinophils, including eosinophil cationic protein, eosinophil-derived neurotoxin and major basic protein. Eosinophil cationic protein and eosinophil-derived neurotoxin exhibit RNase activity and neurotoxicity. Eosinophil-derived neurotoxin induces maturation and activation of DCs by the TLR2-MyD88 signaling pathway and increases Th2 responses [204]. Major basic protein can downregulate the integrity of lipid bilayers [205]. Eosinophils constitutively express IL-31RA and release various proinflammatory cytokines and chemokines, such as IL-1β, IL-6, IL-31, CXCL1, CXCL8, CCL2, CCL18 and CCL26, in response to IL-31 [206,207,208]. IL-31 can prolong the survival of eosinophils by activating ERK signaling [207].

Similar to mast cells, basophils express FcεRI and degranulate after binding of IgE to FcεRI. Basophils play a role in producing Th2 cytokines in response to IL-18, as well as in promoting type 2 innate lymphoid cell (ILC2) recruitment and proliferation by releasing IL-4 in mouse AD models [209]. Basophils express ST2, a receptor for IL-33, and are activated by IL-33 [201]. Basophils express PAMPs, such as NOD2 and TLR2, and play a role in innate immune defense. *S. aureus* exacerbates AD symptoms by binding to NOD2 and TLR2 and by activating basophils and eosinophils in AD mouse models [210]. Recently, NOD2 expression by basophils in AD patients was found to be downregulated, which may explain the ineffective host defense to *S. aureus* in AD [211].

#### 5.2.11. Innate Lymphoid Cells

ILCs are effector cells of innate immunity that are derived from a common lymphoid progenitor, but they do not express cell lineage markers for myeloid and DCs. ILC2s have been detected in the skin, peripheral blood, gastrointestinal tract and airways. ILCs can be divided into three groups based on predominant cytokine type. ILC1s produce Th1 cytokines, including IFN-γ; ILC2s produce Th2 cytokines, including IL-5 and IL-13; and ILC3s produce Th17 cytokines, such as IL-17 and IL-22 [212].

The transcription factor RAR-related orphan receptor alpha and GATA-3 induce differentiation of ILC2s in the presence of IL-25 and IL-33 [213]. Aberrant ILC2 function may contribute to allergic inflammation, such as AD [214,215]. ILC2s express CLA and CCR4, infiltrate the skin after allergen exposure and produce Th2 cytokines. Filaggrin deficiency is associated with increased ILC2 infiltration into the skin in both mice and patients with AD [216]. In response to barrier disruption, keratinocytes produce IL-25, IL-33 and TSLP. ILC2s express IL-25R, ST2 and TSLPR and are activated by IL-25, IL-33 and TSLP to produce IL-5 and IL-13. E-cadherin, the adhesion molecule between keratinocytes, is known to suppress the activation of skin ILC2s, possibly via ligation through killer cell lectin-like receptor G1 on human ILC2s. E-cadherin expression in keratinocytes is reduced due to loss of the skin barrier in AD lesions [217]. Subsequently, ILC2 may contribute to the production of high levels of the Th2 cytokines IL-5 and IL-13 in the absence of the inhibitory E-cadherin signal [213,218]. ILC2 cells produce IL-9, which attracts mast cells into the skin and augments their activation [214]. ILC2s are also activated and migrate in response to prostaglandin D2 [219]. Moreover, depletion of ILC2s blocked the skin homing of Th2 cells by inhibiting the production of the Th2 chemokine CCL17 in DCs in mouse models of AD [214,215,220]. ILC2s stimulated with IL-2 alone were sufficient to drive Th2 responses and AD-like inflammation without the influence of adaptive immunity in mouse models [221,222].

### 5.3. Signal Pathways

#### 5.3.1. GATA-3

GATA-3 is a key regulator of CD4^+^ T cell development, homeostasis, activation and proliferation. GATA-3 is known as a transcription factor that drives the differentiation of Th2 cells, stimulates the secretion of Th2 cytokines from Th2 cells and inhibits the development of B cells. GATA-3 plays a critical role in the differentiation of Th2 cells and is involved in the Th2 cytokine-mediated signaling network in AD. Th2 differentiation could be induced by both STAT6-dependent and STAT6-independent pathways. Acting in the downstream pathway of STAT6, GATA-3 activates IL-4, IL-5 and IL-13, but inhibits the expression of IFN-γ via STAT6-dependent pathways. GATA3 activates the common cellular signaling pathway including c-Jun N-terminal kinases, protein kinase C, JAK-STAT6 and nuclear factor kappa-light-chain-enhancer of activated B cells (NF-κB) in T cells [223]. GATA-3 has a positive-feedback loop that further activates GATA-3 and reinforces Th2 differentiation. In AD lesions, GATA-3 signaling is inhibited by T-bet, which is the Th1 transcription factor. By downregulating STAT-4, GATA-3 enabled Th2-predominant cytokine profiles to be maintained. GATA3 is regulated by diverse upstream signals, including IL-4R/STAT6, IL-2 receptor/STAT5, p38 MAP kinase, T cell receptor and Notch. Notch signaling or IL-2 receptor signaling could induce Th2 differentiation through GATA-3 in the STAT6-independent pathway [224]. Furthermore, AD patients showed a weak T cell receptor signaling, which enhances Th2 immunity and IgE production in order to compensate a weak T cell receptor signaling [225]. 4-hydroxy-3-methoxycinnamaldehyde downregulated T cell proliferation and differentiation into Th1 and Th2 cells by inhibiting T-bet and GATA3, respectively, and could ameliorate the symptoms of AD in mice models [226]. Recently, SB011, a topical DNAzyme that cleaves GATA-3, has been developed and is in a phase II clinical trial [79].

GATA-3 is also involved in Th9 differentiation, but inhibits Th1 and Th17 differentiation. GATA-3 regulates the function of Tregs by inducing forkhead box P3 (Foxp3) expression. As mentioned above, GATA-3 plays important roles in the generation and function of ILCs [223]. A recent GWAS of severe AD found new loci including candidate genes that may contribute to the defects in GATA-3 and STAT6 [59,227,228].

B cell activation is initiated by the engagement of B cell receptor and co-receptor, which leads to the change in the gene expression, as well as common cellular signaling, such as protein kinase C, NF-κB and c-fos. Information regarding unique abnormalities of B cell signaling in the AD pathogenesis is not well known. In order to establish B cell generation and maturation, CD40-CD40L interaction between B cells and T cells is essential, and B cell activation is parallel to T cell activation in a thymus-dependent manner [229].

#### 5.3.2. Notch Signaling

The lesional epidermis of patients with AD exhibits markedly suppressed expression of Notch and its receptors, and reduced Notch expression in the lesions was shown to be normalized after successful treatment [230]. Impaired Notch signaling adversely influences epidermal terminal differentiation by affecting filaggrin, involucrin and transglutaminase-3 activity, resulting in incomplete CE and barrier formation. Suppressed Notch signaling downregulates the expression of aquaporin 3 and claudin-1, thus resulting in barrier dysfunction and increased transepidermal water loss [230,231]. Notch inhibits TLR-activated innate immunity by a negative feedback mechanism [232]. Thus, deficiency in Notch signaling may result in persistent activation of macrophages and DCs as observed in AD. The IFNG has recently been found as a target of Notch1 [233]. Compromised Notch signaling may thus explain the increased susceptibility to viral skin infection in AD through diminished IFN-γ production. Notch1 is an inhibitor of a transcription factor called activator protein-1, which is upregulated in the AD-affected epidermis and promotes Th2 cytokine production. The absence of Notch-mediated downregulation of activator protein-1 results in upregulation of the levels of IL-31 and may aggravate IL-31-mediated pruritus in AD [231]. Notch deficiency induces keratinocytes to secrete TSLP, which has recently been shown to stimulate cutaneous sensory neurons to promote itch [234]. Notch-knockout mice exhibit AD-like skin inflammation. In the presence of TGF-β, Notch1 regulates Foxp3 expression, which is important in the differentiation of Tregs.

## 6. Conclusions

**Figure 1:**
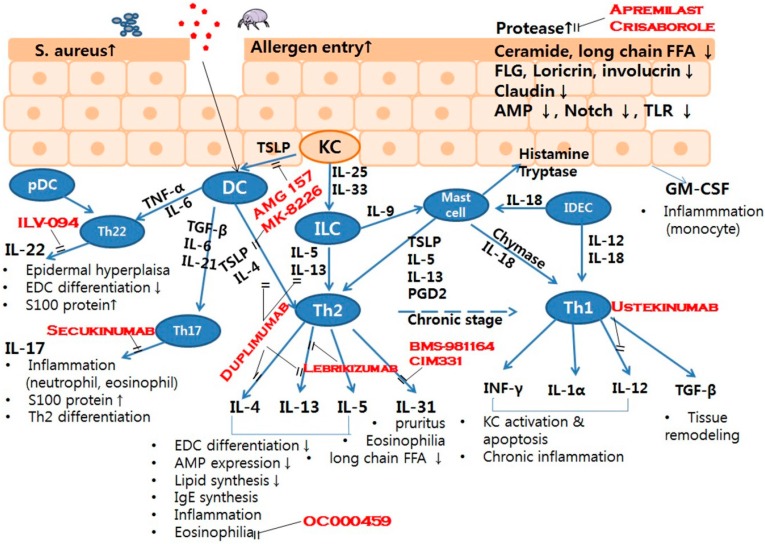
Barrier and immune network in the pathogenesis of atopic dermatitis. Red words: drug name; Dotted line (chronic stage): disease progression; Line: secretion.

Patients with AD have impaired skin integrity and show elevated susceptibility to allergens and pathogens, which activate innate and adaptive immune responses. AD is characterized as a Th2/Th22-predominant inflammatory disease, but Th1 and Th17 responses modulate the development and progression of AD. Although T cells play key roles in the inflammation seen in AD, keratinocytes, DCs, B cells, mast cells, eosinophils, basophils and ILC2s act together through various cytokines and chemokines (Figure 1).

Therapeutics include: duplimumab, anti-IL-4Ra monoclonal antibodies (mAb); lebrikizumab, anti-IL-13 mAb; ILV-094, anti-IL-22 mAb; BMS-981164, anti-IL-31 mAb; CIM331, anti-IL-31R mAb; AMG 157, anti-TSLP mAb; MK-8226 anti-TSLPR mAb; ustekinumab, anti-IL-12/IL-23 mAb; secukinumab, anti-IL-17 mAb; OC000459, chemoattractant receptor-homologous molecule expressed on Th2 cell antagonist.

IL-4, -5, -13 and -31 drive Th2 inflammatory responses, but also inhibit epidermal terminal differentiation and lipid barrier formation and, thus, disrupt barrier functions. This is consistent with the “outside-in” and “inside-out” hypotheses [235]. Complex interactions between genes and environmental factors have been revealed, and further studies will reveal how environmental changes can regulate the development and triggering of AD. Current therapeutic options are often unsatisfactory to the refractory AD patients. Taken together, these data extend our knowledge of newly-found genes and signaling molecules as possible therapeutic targets for AD.

## Figures and Tables

**Table 1:** Susceptibility genes for skin barrier and immunity in atopic dermatitis.

Gene	Locus	Alleles or Mutation	SNP	Population	Disease Severity	Reference
**Epidermal Differentiation Complex**						
*FLG*	1q21.3	Loss-of-function Copy number variation	R501X, 2282del4	European, Chinese, Singaporean	Increases the risk of AD correlated with severity	[14,16,18,19,20,21,22]
*FLG2*	1q21.3	Premature stop codon	rs12568784, Q2053del224 rs16833974	African American	More persistent AD	[23]
*SPINK5*	5q31	Loss-of-function	rs2303070 T	Taiwanese	Increases the risk of AD	[30]
			E420K	Italian	Increases the risk of AD	[31]
*SPRR3*	1q21.3	Copy number variation	rs28989168	German	Increases the risk of AD	[25]
*TMEM79*		Missense mutation	rs6684514	Ireland	Increases the risk of AD	[27]
*claudin-1*	3q28	Haplotype-tagging	rs893051	African American	Increases the risk of AD	[32]
		AG or GG genotype	rs9290929	Korean	Mold infection	[33]
**Innate Immunity**						
*TLR2*	4	Missense mutation	R753Q	German, Italian	Severe AD	[40]
		A	16934T	German, Japanese	Severe AD	[41,42]
*TLR4*	9	N/A	D299G	Italian	Increased in AD	[43]
		896A/G	N/A	Ukrainian	Increased viral respiratory infections	[44]
*TLR9*	3	TT	C-1237T	German	Intrinsic AD	[45]
*NOD1*		N/A	rs2907748 rs2907749	German	Allergen sensitization	[46]
*hBD 1*	8	haplotype CT	rs5743399	Korean	allergen sensitization	[47]
		N/A	rs5743409	Korean	AD	[47]
*TSLP*	5q22	C/T	rs1898671	European American	Eczema herpeticum	[48]
**Adaptive Immunity**						
*IL-18*	11q22	G-allele	rs1946518 rs187238	Chinese	Low risk of AD	[51]
*IL18RAP*	2q12	N/A	rs6419573	Japanese	Increase the risk of AD	[35]
*IL-12*	chr3	IVS-798A/T, haplotype TA	rs582504, rs582054, rs2243151	Korean	Increase the risk of AD	[53]
*IL-12RB*	chr5 chr1	TT AA	rs438421, rs2066446	Korean	Allergen sensitization	[53]
*IFNG/IFNGR1*	12/6q23-24	Loss-of-function	V14M and Y397C	African American	Eczema herpeticum	[52]
*IL-4*	5q31-33	T allele	590 C/T of IL-4 promoter	Egyptian	Increase the risk of AD	[58]
*IL-4Rα*	16	Gain of function	I50V, Q576R	Egyptian	Increase the risk of AD	[58]
*IL-13*	5q31.1	N/A	rs12188917		Association with asthma	[34]
*STAT6*	12	Minor allele homozygotes	rs324011	German	Low risk of AD	[59]
*IL-31*	12	Haplotype AAA and GAA	1066, −2057, and ivs2 + 12	polish	High IL-31 serum level severe pruritus	[60]
*IL-17A*		AA genotype	152 G/A	polish	Severe AD in coexistence of asthma	[61]
*FCER1A*	1	N/A	promoter	Japanese	Allergen sensitization	[74]
**Chemokines**		N/A				
*RANTES*	17.35	28G	N/A	German	Allergen sensitization	[69]
		403A overexpression	N/A	Japanese, German	Allergen sensitization	[70]
**Vitamin D Pathway**						
*Cyp24a1*	20q54	Major C allele	rs2248359	German	Severe AD	[55]
*VDR*	20q13	AT	rs7975232	Chinese	Severe, eosinophilia and high IgE levels	[54]
**Nerve Growth Factor Pathway**						
BDNF	11	T	C270T	Chinese	Intrinsic AD and male sex	[77]

N/A: not available.

## Abbreviations

AD	Atopic dermatitis
AMPs	Antimicrobial peptides
CARD	Caspase recruitment domain
CCL	C-C motif chemokine ligand
CCR	C-C motif chemokine receptor
CLDN1	Claudin-1
CE	Cornified envelope
CXCL	C-X-C motif chemokine ligand
DCs	Dendritic cells
EDC	Epidermal differentiation complex
ERK	Extracellular signal-regulated kinase
FcεRI	High affinity IgE receptor
FFA	Free fatty acid
FLG	Filaggrin gene
Foxp3	Forkhead box P3
GATA-3	GATA-binding protein 3
GWAS	Genome-wide association study
hBD	Human β-defensin
HRH4	Histamine receptor H4
IDECs	Inflammatory dendritic epidermal cells
IFNG	*IFN-γ* gene
ILCs	Innate lymphoid cells
*IL18RA* *P*	Interleukin 18 receptor accessory protein
JAK	Janus kinase
JNK	c-Jun *N*-terminal kinase
LCs	Langerhans cells
LEKTI	Lymphoepithelial Kazal-type trypsin inhibitor
mAb	Monoclonal antibodies
miR	MicroRNA
MMP	Matrix metalloproteinase
NALP	NACHT, LRR and PYD domain-containing protein
NF-κB	Nuclear factor κ-light-chain-enhancer of activated B cells
NOD	Nucleotide-binding oligomerization domain receptors
PAR2	Protease-activated type 2 receptor
PDE4	Phosphodiesterase 4
RANTES	Regulated on activation, normal T cell expressed and secreted
SC	Stratum corneum
SNP	Single nucleotide polymorphism
SP	Serine protease
STAT	Signal transducer and activator of transcription
Th	T helper
TLR	Toll-like receptor
TMEM79	Transmembrane protein 79
Treg	Regulatory T cell
TSLP	Thymic stromal lymphopoietin
SPINK5	Serine protease inhibitor Kazal-type 5
SPRR3	Small proline-rich protein 3
